# Failure to filter: anxious individuals show inefficient gating of threat from working memory

**DOI:** 10.3389/fnhum.2013.00058

**Published:** 2013-03-04

**Authors:** Daniel M. Stout, Alexander J. Shackman, Christine L. Larson

**Affiliations:** ^1^Department of Psychology, University of Wisconsin - MilwaukeeMilwaukee, WI, USA; ^2^HealthEmotions Research Institute and Lane Neuroimaging Laboratory, Wisconsin Psychiatric Institute and Clinics, University of Wisconsin - MadisonMadison, WI, USA

**Keywords:** anxiety disorders, attention, contralateral delay activity (CDA), emotion-cognition interactions, event-related potential (ERP), individual-differences, trait anxiety, working memory

## Abstract

Dispositional anxiety is a well-established risk factor for the development of psychiatric disorders along the internalizing spectrum, including anxiety and depression. Importantly, many of the maladaptive behaviors characteristic of anxiety, such as anticipatory apprehension, occur when threat is absent. This raises the possibility that anxious individuals are less efficient at gating threat's access to working memory, a limited capacity workspace where information is actively retained, manipulated, and used to flexibly guide goal-directed behavior when it is no longer present in the external environment. Using a well-validated neurophysiological index of working memory storage, we demonstrate that threat-related distracters were difficult to filter on average and that this difficulty was exaggerated among anxious individuals. These results indicate that dispositionally anxious individuals allocate excessive working memory storage to threat, even when it is irrelevant to the task at hand. More broadly, these results provide a novel framework for understanding the maladaptive thoughts and actions characteristic of internalizing disorders.

## Introduction

Anxiety disorders are debilitating, highly prevalent, and associated with substantial morbidity and mortality (Sareen et al., 2005; Collins et al., 2011; Kessler et al., 2012; Taylor et al., 2012). High levels of dispositional anxiety and behavioral inhibition are a well-established risk factor for anxiety, depressive, and other psychiatric disorders (Lahey, 2009; Kotov et al., 2010; Blackford and Pine, 2012; Clauss and Blackford, 2012), highlighting the importance of understanding the neurocognitive underpinnings of this key risk factor. Indeed, alterations in core cognitive processes, such as executive control and working memory, are central to neurocognitive theories of anxiety (Bishop, 2007, 2008; Eysenck et al., 2007; Eysenck and Derakshan, 2011; Berggren and Derakshan, in press).

Difficulties controlling the processing of threat are a central feature of dispositional anxiety and the anxiety disorders; anxious individuals frequently allow threat-related information to unduly control their thoughts and actions. In particular, there is considerable evidence that anxious individuals are biased to allocate excess attention to threat-related cues when they are present in the immediate environment (e.g., words, faces; Cisler and Koster, 2010), even when this comes at the expense of task-goals and on-going behavior (Bishop et al., 2004, 2007; Etkin et al., 2009). This attentional bias to threat has been proposed to be a specific causal risk factor for the development and maintenance of anxious psychopathology (Bar-Haim et al., 2007; Hofmann et al., 2012; MacLeod and Mathews, 2012; Shechner et al., 2012).

Importantly, many of the maladaptive thoughts and actions characteristic of anxious individuals occur when threat-related cues are absent from the immediate external environment (e.g., anticipatory apprehension, behavioral avoidance, and intrusive thoughts)—a key clinical feature that is not addressed by research focused on attentional biases to threat cues. This raises the possibility that dispositional anxiety reflects a broader regulatory deficit that encompasses problems governing threat's access to working memory. Working memory is the “blackboard of the mind” (Goldman-Rakic, 1996, p. 13473), a limited capacity workspace where information is actively maintained, recalled, and manipulated (Cowan, 2005; Baddeley, 2012). The internal representation of task sets and other kinds of goals in working memory plays a critical role in sustaining goal-directed attention, information processing (e.g., memory retrieval), and action in the face of competition with potential sources of distraction or interference (Miller and Cohen, 2001; Postle, 2006; D'Ardenne et al., 2012). This framework suggests that the maladaptive cognitive-behavioral profile characteristic of anxious individuals reflects a failure to prevent threat from gaining access to working memory. Allowing threat-related distracters access to working memory would potentially allow them to bias the stream of information processing after they are no longer present in the external environment. Ultimately, the unnecessary entry of threat into working memory may promote worry, intrusive thoughts, and other anxiety-related cognitions that disrupt on-going behavior (Thiruchselvam et al., 2012).

Here, we used a well-validated neurophysiological measure of working memory storage, *contralateral delay activity* (CDA; Vogel and Machizawa, 2004), to directly test whether dispositionally anxious individuals have difficulty preventing threat-related distracters from gaining access to working memory. The amplitude of the CDA, an event-related potential (ERP) that persists throughout the retention period of visual working memory tasks, is highly sensitive to the number of items maintained in working memory (Vogel and Machizawa, 2004; McCollough et al., 2007; Ikkai et al., 2010; Voytek and Knight, 2010). We measured CDA during a working memory task in which subjects were instructed to selectively retain one or more emotional faces while ignoring others (Sessa et al., 2011). Faces were either threat-related (i.e., fearful; Whalen, 1998; Davis and Whalen, 2001) or emotionally-neutral. This procedure allowed us to quantify the number of task-irrelevant distracter faces that gained access to working memory, indexed by increased CDA amplitude (Vogel et al., 2005). Critically, it also made it possible to measure the extent to which higher levels of dispositional anxiety, measured using the well-validated State-Trait Anxiety Inventory (STAI; Spielberger et al., 1983), are associated with problems gating threat-related distracters from working memory.

## Methods

### Subjects

Thirty-four (22 female) students from the University of Wisconsin, Milwaukee community participated in exchange for course extra-credit (*M* = 21.83 years, *SD* = 5.34). Subjects provided written informed consent prior to the experiment. The study was approved by the University of Wisconsin, Milwaukee's Institutional Review Board. One subject was removed due to chance performance. Nine subjects were excluded from analyses due to excessive ocular artifacts, a rate that is consistent with prior research using similar tasks (e.g., ~35%; Sessa et al., 2011). A total of 24 subjects remained for further analysis.

### Quantifying dispositional anxiety

All subjects completed the trait version of the STAI (Spielberger et al., 1983), a 20-item measure of trait or dispositional anxiety (e.g., *Some unimportant thought runs through my mind and bothers me*, *I take disappointments so keenly that I can't put them out of my mind*, *I worry too much over something that really doesn't matter*). The STAI has been shown to exhibit high internal-consistency reliability (α = 0.89) and test-retest stability (*r* = 0.88; Barnes et al., 2002). The distribution of scores in the present sample (*M* = 38.2, *SD* = 9.43, range of 20–53) was similar to published norms for mixed-sex undergraduate populations (Spielberger et al., 1983).

### Working memory task

**Figure 1:**
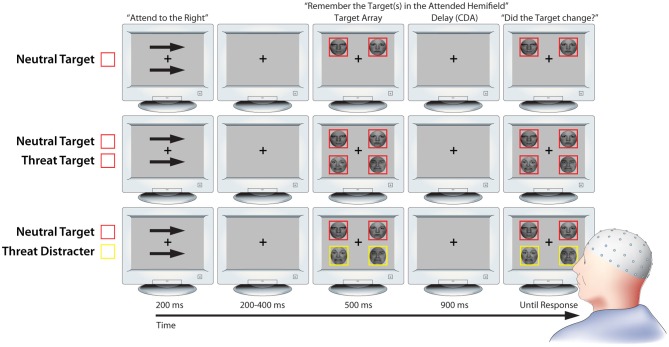
**Working memory task.** Rows depict three key conditions from the lateralized change detection (i.e., working memory) task (from top to bottom: NT1, NT1FT1, NT1FD1). As detailed in the Methods section, lateralized presentation was necessary for isolating contralateral delay activity (CDA). Attention was directed to one hemifield by the arrow cues; identical stimuli were presented in the uncued hemifield to control for non-specific perceptual and preparatory motor activity when calculating CDA. Delay-spanning CDA was extracted from the 900 ms delay epoch. For ease of interpretation, the schematic is not to scale. Portions of this figure were reprinted by permission from Macmillan Publishers Ltd.: *Nature Reviews Neuroscience* (Houdé and Tzourio-Mazoyer, 2003; Peelen and Downing, 2007).

We used a lateralized change detection task to estimate the number of threat-related (i.e., fearful) and emotionally-neutral faces stored in working memory, as indexed by the CDA. As detailed below, the use of lateralized stimulus displays was mandated by our focus on CDA (Figure 1; Vogel and Machizawa, 2004; Perez and Vogel, 2012). The trial sequence was adapted from a report by Sessa et al. (2011) and began with a fixation-cross (500 ms). Next, a pair of arrows indicating the to-be-remembered hemifield was presented above and below the fixation-cross (200 ms). Following a brief interstimulus interval (200–400 ms), an array of 2 or 4 faces was presented (500 ms). Participants were instructed to attend to one or two target faces, which were surrounded by red (or yellow) borders in the cued hemifield, and to ignore distracter faces, which were surrounded by yellow (or red) borders. The pairing of colors with targets or distracters was counterbalanced across participants.

CDA was quantified during the subsequent retention period (900 ms). This was followed by a probe array. Subjects were instructed to make a response indicating whether or not a target face had changed identity (equiprobable; button contingencies counterbalanced across subjects). The probe array was presented until a response was registered. On change trials, the identity of one of the target faces changed while the expression remained invariant. The fixation-cross was displayed during inter-trial intervals (800–1200 ms). Set-sizes of 1 and 2 were used because previous research has shown that working memory capacity saturates at approximately 2 faces (Jackson and Raymond, 2008; Jackson et al., 2009).

### Design

To assess the influence of expression and individual differences in anxiety on the ability to prevent task-irrelevant faces from entering working memory, the task included conditions in which threat-related distracters (1 Neutral Target and 1 Fear Distracter [NT1FD1]) or neutral distracters were present (1 Neutral Target and 1 Neutral Distracter [NT1ND1]). These conditions allowed us to calculate “filtering efficiency” scores (detailed below; Jost et al., 2011), reflecting the degree of unnecessary storage, for each expression. To confirm that CDA was sensitive to the number of faces retained in working memory, the task also included conditions in which set size was varied and only task-relevant targets were presented (i.e., 1 Neutral Target [NT1], 2 Neutral Targets [NT2], 1 Fear Target [FT1], 2 Fear Targets [FT2], and 1 Neutral Target paired with 1 Threat Target [NT1FT1]). Subjects completed 32 practice trials before beginning the experimental trials, which included 180 trials/condition for a total of 1260 trials organized into twenty 63-trial blocks. The condition order was pseudo-randomized across blocks within-subjects.

### Face stimuli

Face stimuli consisted of 52 black-and-white images (26 unique models; half expressing fear) from the MacBrain Face Stimulus Set (http://www.macbrain.org/faces) or Ekman and Friesen's (1976) set. Images were digitally manipulated to remove non-face features (e.g., hair, clothing) and equate luminance. Faces were presented in rectangular borders (2.4° wide × 2.8° tall) at a viewing distance of ~65 cm. Both the memory array and probe array contained faces that were placed in fixed locations surrounding a fixation cross. Horizontal distance between the face stimuli and the fixation cross was 3°. Vertical distance between top and bottom face was 1.5°.

### ERP data acquisition and preprocessing

ERPs were recorded using a DC amplifier and a 32-channel cap with shielded leads (Advanced Neuro Technology B.V., Netherlands) referenced to the left mastoid. Impedances were kept below 10 kΩ. Data were low-pass filtered (~69.12 Hz) and sampled at 256 Hz. The vertical electrooculogram (VEOG) was measured using a pair of bipolar-referenced electrodes placed above and below the right eye. The horizontal electrooculogram was recorded using a pair of bipolar-referenced electrodes placed 1 cm from the outer canthi of the eyes.

Offline, ERP data were re-referenced (mean of the left and right mastoids), filtered (Butterworth band-pass of 0.1–30 Hz; 24db/octave), segmented (–200 to 1400 ms from the onset of the target array), and baseline-corrected (200 ms). Because the CDA critically depends on lateralized visual processing, we elected to reject all trials in which there was evidence that subjects failed to attend to the center of the visual field, rather than use artifact-correction algorithms that could potentially mask shifts in visual attention (Shackman et al., 2009; McMenamin et al., 2010, 2011). Accordingly, trials where VEOG exceeded ±80 μV and/or other channels exceeded ±60 μV were automatically rejected. Nine subjects with excessive artifact (>35% trials) were excluded from analyses, consistent with other studies using similar tasks (e.g., Sessa et al., 2011). For the remaining subjects, an average of 79.87% (*SD* = 0.08) of trials were retained. Importantly, the retained and excluded subjects did not significantly differ in either the mean level of dispositional anxiety or estimated working memory capacity, *t*s < 0.68, *p*s > 0.51.

### CDA

To isolate CDA, contralateral waveforms were created by averaging the activity recorded in the left hemisphere when attending to cued stimuli in the right visual field, and activity over the right hemisphere when attending to cued stimuli in the left visual field. Ipsilateral waveforms were created by averaging the activity recorded in the left hemisphere when attending to uncued stimuli in the left visual field, and activity over the right hemisphere when attending to uncued stimuli in the right visual field (see Figure 1). CDA was calculated as the difference between contralateral and ipsilateral activity during the retention interval (500–900 ms; Figure 1). In contrast to other neurophysiological measures of delay-spanning activity, these procedures for isolating CDA have the advantage of removing nonspecific perceptual (i.e., elicited by physically-identical stimuli in the uncued visual field) and motor preparatory activity (Vogel and Machizawa, 2004; Vogel et al., 2005). Averaged waveforms were created for each condition and hemisphere using electrode clusters (P3/4, P7/8, O1/2, and T7/8). Consistent with prior work, error trials were excluded when calculating CDA for the conditions in which only targets were presented (Vogel et al., 2005), but were not excluded when calculating CDA for the conditions in which a mixture of targets and distracters was presented (Lee et al., 2010). Error trials were used for the mixed conditions because decrements in performance likely reflect the storage of distracters in working memory (Lee et al., 2010). For visualization purposes, grand averaged waveforms were low-pass filtered (10 Hz).

### Confirmatory analyses

To confirm that task-relevant threat-related targets are associated with enhanced storage (Sessa et al., 2011) and that larger target arrays (i.e., set sizes) are associated with increased working memory storage, we performed a series of analyses using CDA, as well as behavioral estimates of working memory capacity and reaction time (RT). Working memory capacity was estimated using Pashler's (1988) formula: *K* = S × (*H* − FA)/(1 − FA), where *K* is the estimated number of items maintained in WM, *S* is the set-size of the memory array, *H* is the hit-rate, and *FA* is the false alarm rate. Pashler's *K* was used because it was developed for working memory tasks using whole-display probes; whereas the more commonly used Cowan's *K* (Cowan, 2001) was developed for single-probe displays (see Rouder et al., 2011 for a detailed discussion). Analyses were performed using SPSS (version 18.0.0; IBM Inc., Armonk, NY).

### Hypothesis testing (filtering efficiency)

To test whether dispositionally anxious individuals fail to regulate threat's access to working memory, CDA “filtering efficiency” scores (Jost et al., 2011) were separately computed for the threat and neutral distracter conditions. Filtering efficiency for threat-related distracters was calculated as the difference in amplitude between trials in which two targets were presented (1 Neutral Target and 1 Fear Target [NT1FT1]) and physically-identical trials in which a neutral target was paired with a fear distracter (NT1FD1). Because CDA is a negative-going potential, difference scores were scaled by −1 to aid interpretation. An efficiency of zero indicates a complete failure of filtering (i.e., equivalent storage of two targets compared to the combination of a target and a threat-related distracter). Likewise, filtering efficiency for neutral distracters was calculated as the difference in amplitude between trials in which two neutral targets (NT2) were presented and trials in which a neutral target was paired with a neutral distracter (NT1ND1) (scaled by –1).

Hypothesis testing on relations between dispositional anxiety (i.e., STAI) and filtering efficiency was performed using a series of regressions. A single outlier was excluded from the analyses of neutral filtering efficiency. Results were similar with the outlier included (not reported). To assess the specificity of relations between dispositional anxiety and CDA filtering efficiency, we computed additional regressions controlling for nuisance variation in mean-centered age, sex, and maximum working memory capacity (i.e., the maximal Pashler's *K* across any of the five “pure” target conditions). Robust regressions, which minimize the influence of outlying observations (e.g., Shackman et al., in press; Wager et al., 2005), yielded equivalent results. Although hypothesis testing focused on CDA filtering efficiency, exploratory analyses of RT filtering efficiency were also performed. RT filtering efficiency was computed using the same formulas described for CDA, but without the −1 scalar.

## Results

### Threat-related targets are associated with enhanced storage

**Figure 2:**
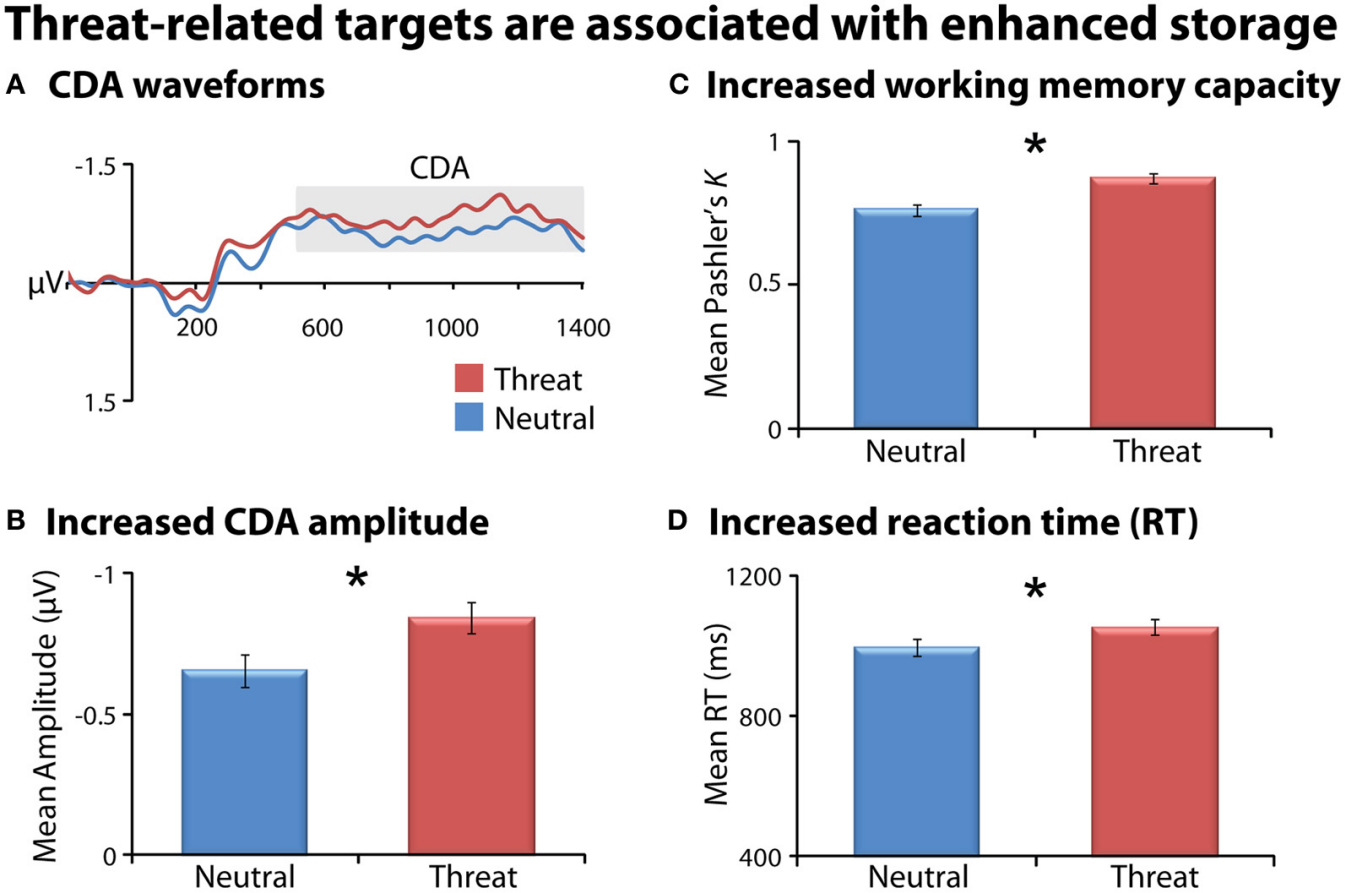
**Task-relevant threat targets are associated with enhanced storage.** Means are collapsed across set-size (NT1/NT2 and FT1/FT2). Contralateral delay activity (CDA) waveforms (panel **A**). Mean CDA amplitude was extracted using the entire delay interval (500–1400 ms; gray box). Threat (red) was associated with increased CDA amplitude (panel **B**), working memory capacity (panel **C**), and reaction time (RT; panel **D**) compared to neutral (blue). Asterisks denote significant pairwise mean differences (*p* < 0.05). Error bars indicate the nominal probability of the null hypothesis being rejected by chance: *p* < 0.05 (non-overlapping bars) or *p* > 0.05 (overlapping bars). Bars were computed as described in Shackman et al. (2010). Note that for CDA results, negative is plotted up corresponding to increased amplitude.

As a precursor to hypothesis testing, we examined the influence of threat on working memory storage when it is task-relevant. Consistent with previous research (Sessa et al., 2011), task-relevant threat targets (FT1, FT2) were associated with enhanced storage compared to emotionally-neutral targets (NT1, NT2), evidenced by enhanced CDA, increased *K*, and slower responses (*F*s_(1, 23)_ > 6, *p*s < 0.03; Figure 2 and Table 1). As expected, larger target arrays were associated with increased storage, as indexed by the same three measures (*F*s_(1, 23)_ > 6.3; *p*s < 0.03).

**Table 1:** **Means and standard deviations for accuracy, working memory capacity (*K*), and reaction time (in milliseconds) for each condition**.

**Condition**	**Accuracy (proportion correct)**	**Working memory capacity (*K*)**	**RT (ms)**
1 Neutral target	0.83 (0.10)	0.74 (0.17)	900.65 (179.42)
2 Neutral targets	0.66 (0.07)	0.78 (0.35)	1108.33 (268.45)
1 Fear target	0.86 (0.09)	0.79 (0.16)	976.05 (209.37)
2 Fear targets	0.70 (0.07)	0.96 (0.32)	1139.68 (260.42)
1 Neutral target and 1 Fear target	0.69 (0.08)	0.92 (0.34)	1104.41 (251.72)
1 Neutral target and 1 Fear distracter	0.80 (0.11)	0.70 (0.19)	995.99 (215.20)
1 Neutral target and 1 Neutral distracter	0.79 (0.10)	0.69 (0.17)	993.00 (187.31)

### Inefficient filtering of threat-related distracters

**Figure 3:**
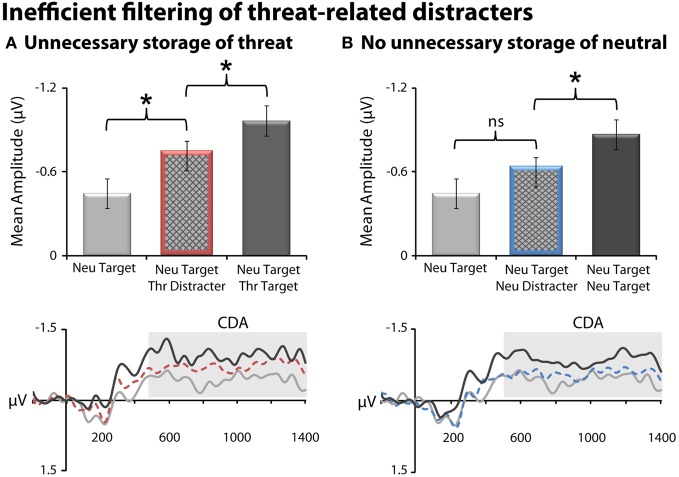
**Threat-related distracters were inefficiently filtered from working memory, as indexed by contralateral delay activity (CDA). (A)** Threat distracters. Mean CDA amplitude was significantly increased (i.e., more negative) on trials with a threat-related distracter (red bar) (NT1FD1) compared to those with a single neutral target (light gray) (NT1). On average, subjects were able to filter threat-related distracters, albeit inefficiently; mean CDA amplitude was significantly decreased on trials with a threat-related distracter (NT1FD1) compared to those with two physically-matched targets (dark gray) (NT1FT1). CDA waveforms for the three conditions are shown at the bottom. Mean CDA amplitude was extracted using the entire delay interval (500–1400 ms; gray box). **(B)** Neutral distracters. Mean CDA amplitude was not significantly increased on trials with a neutral distracter (blue bar) (NT1ND1) compared to those with a single neutral target (light gray) (NT1). Asterisks denote significant pairwise mean differences (*p* < 0.05). Error bars indicate the nominal probability of the null hypothesis being rejected by chance: *p* < 0.05 (non-overlapping bars) or *p* > 0.05 (overlapping bars). Bars were computed as described in Shackman et al. (2010). Note that negative potentials are plotted up corresponding to increased CDA amplitude.

Threat-related distracters gained unnecessary access to working memory, as indexed by increased CDA amplitude for the threat-distracter condition (NT1FD1) compared to a single neutral target (NT1), *t*_(23)_ = 2.40, *p* = 0.03 (Figure 3). On average, subjects were able to filter threat-related distracters, albeit inefficiently. Specifically, the amplitude of CDA was significantly smaller for the threat-distracter condition (NT1FD1) compared to those in which two targets were presented (NT1FT1), *t*_(23)_ = −3.61; *p* = 0.001. Unlike threat, neutral-distracters were efficiently filtered; CDA amplitude did not differ between the neutral-distracter (NT1ND1) and single target conditions (NT1), *t*_(23)_ = 1.4; *p* = 0.18 (Figure 3) but was significantly smaller than the two neutral target condition (NT2), *t*_(23)_ = −2.61, *p* = 0.02.

### Anxious individuals fail to filter threat-related distracters

**Figure 4:**
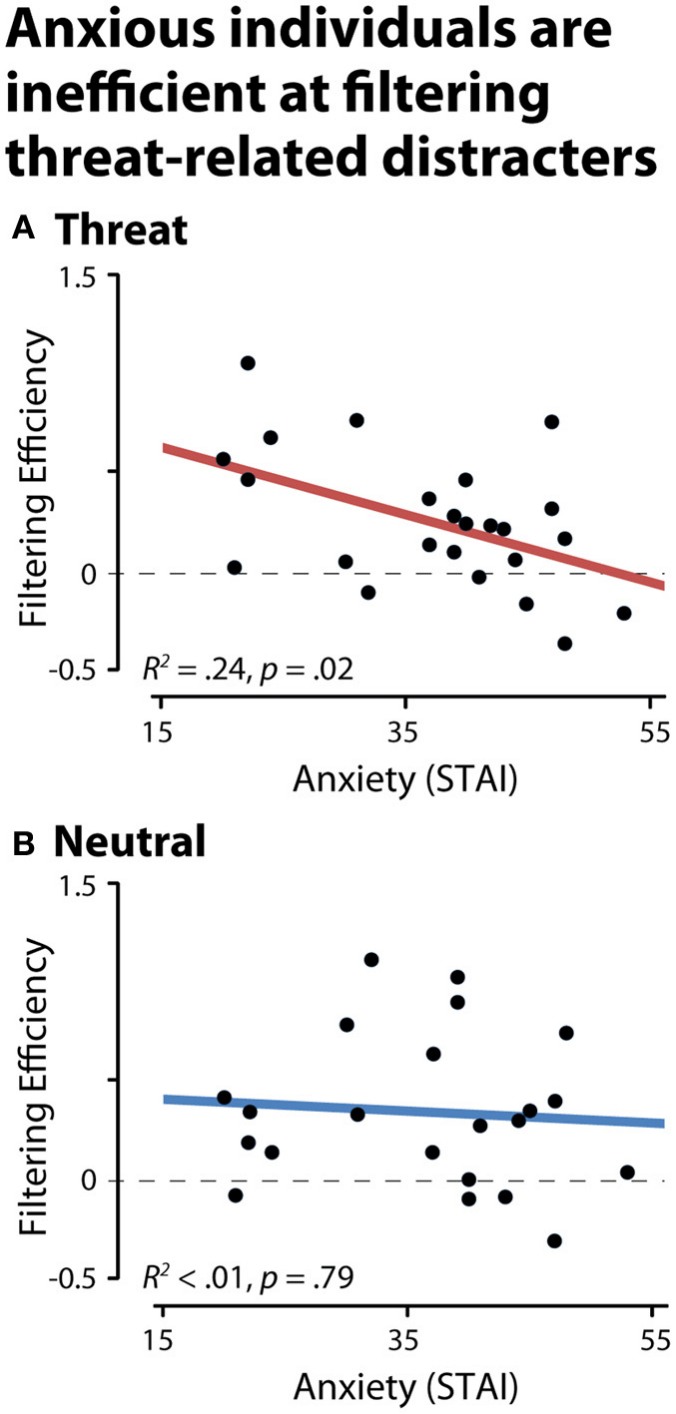
**Dispositionally anxious individuals are inefficient at filtering threat distracters, as indexed by contralateral delay activity (CDA). (A)** Threat-related distracters. **(B)** Neutral distracters. A filtering efficiency of zero (broken gray line) indicates a complete failure of filtering (i.e., comparable levels of storage in the distracter and two-target conditions, NT1FT1—NT1FD1 and NT2-NT1ND1).

To test whether anxious individuals exhibit difficulties gating threat-related distracters from working memory, we used the CDA to compute filtering efficiency scores (see the Methods section; Jost et al., 2011). An efficiency of zero indicates a complete failure of filtering, that is, comparable levels of storage in the physically-identical distracter and two-target conditions. Analyses of CDA filtering efficiency demonstrated that anxious individuals were less efficient at preventing threat-related distracters from gaining access to working memory, *R*^2^ = 0.24, *p* < 0.03 (Figure 4). Similar effects were obtained after controlling for nuisance variation in age, sex, and maximum working memory capacity (partial *R*^2^ > 0.31, *p* < 0.01) or the number of artifact-free trials contributing to the CDA analyses (partial *R*^2^ = 0.20, *p* = 0.03). Dispositional anxiety was unrelated to the efficiency of filtering emotionally-neutral distracters (*R*^2^ < 0.01, *p* > 0.05). To confirm that our results were not unduly influenced by outlying values, we recomputed the key analyses using robust regression techniques. This revealed nearly identical results: higher levels of dispositional anxiety predicted reduced efficiency for filtering threat-related distracters (*R*^2^ = 0.25, *p* < 0.01), but not neutral distracters (*R*^2^ < 0.01, *p* > 0.05).

Likewise, dispositional anxiety did not predict CDA amplitude when threat-related targets were relevant (FT1 and FT2) to the task, *R*^2^ < 0.02, *p* > 0.05. Consistent with these results, anxiety significantly predicted threat filtering efficiency after controlling for either variation in neutral filtering efficiency or the CDA associated with task-relevant threat targets (partial *R*^2^ > 0.24, *p*s < 0.05). Exploratory analyses of RT filtering efficiency revealed a similar pattern. Specifically, higher levels of dispositional anxiety predicted reduced filtering efficiency for threat-related (*R*^2^ = 0.22, *p* = 0.02), but not neutral distracters (*R*^2^ < 0. 01, *p* > 0.05). Maximum working memory capacity did not predict filtering efficiency for either the threat or neutral distracter conditions (*R*^2^s < 0.03, *p*s < 0.05), likely reflecting the rather limited variation in capacity for faces (Jackson and Raymond, 2008; Jackson et al., 2009; Sessa et al., 2011).

## Discussion

The present results provide compelling new evidence that dispositionally anxious individuals allocate unnecessary working memory storage to threat-related cues when they are irrelevant to the task at hand. This effect was not evident for emotionally-neutral distracters and could not be explained by individual differences in working memory capacity, the size of the CDA evoked by task-relevant threat targets, or the efficiency of filtering emotionally-neutral distracters. Parallel results were obtained for RT. Taken together these data indicate that dispositional anxiety is associated with a specific deficit in preventing threat-related distracters from gaining access to working memory. These results reinforce work emphasizing the importance of cognitive control deficits in anxiety and mood disorders (Eysenck et al., 2007; Eysenck and Derakshan, 2011; Owens et al., 2012). More generally, our results provide a novel neurobiological framework for conceptualizing the neural mechanisms that underlie the intrusive thoughts and maladaptive actions characteristic of anxious individuals when threat is absent.

Our findings demonstrate that anxiety is associated with inefficient gating of threat-related distracters from working memory, but they do not directly address the neural mechanisms underlying this deficit. Prior work using simple geometric stimuli suggests that CDA reflects the activity of a capacity-limited buffer instantiated in the posterior parietal cortex (PPC; Todd and Marois, 2004, 2005; Xu and Chun, 2006). Presently, the specific neural mechanisms underlying anxious individuals' inability to adequately gate threat's access to this buffer remain unknown. Our results are compatible with alterations in any of three distinct functional circuits. A key challenge for future research will be to directly test these hypotheses.

One possibility is that the unnecessary storage of threat-related distracters in PPC reflects the amygdala's influence on the visual cortical regions responsible for processing threat-related cues, such as the faces used in our study. Among anxious and behaviorally inhibited individuals, the amygdala is more reactive to potential threat (Schwartz et al., 2003; Etkin and Wager, 2007; Blackford et al., 2012). The amygdala is poised to bias attention to threat via excitatory projections to the visual cortex (Vuilleumier et al., 2004; Freese and Amaral, 2009). Indeed, functional connectivity between these two regions is increased when attending to threat cues (Noesselt et al., 2005; Mohanty et al., 2009) and threat-induced recruitment of the amygdala precedes enhanced activation of visual cortex (Sabatinelli et al., 2009; Pourtois et al., in press). Variation in amygdala activation also predicts the reorienting of attention to threat-related cues (Gamer and Büchel, 2009) and the trial-by-trial detection of threat—an effect mediated by activation in the visual cortex (Lim et al., 2009). Collectively, these data suggest that difficulties regulating threat's access to working memory could be a downstream consequence of anxious individuals' bias to over-allocate covert and overt attention to threat (Bar-Haim et al., 2007).

A second possibility is that the unnecessary occupation of working memory by threat reflects problems monitoring the competition between targets and threat-distracters for attention. Adjudication of this competition is thought to depend upon conflict-monitoring processes instantiated in the midcingulate cortex (MCC; Botvinick, 2007; Shackman et al., 2011). When conflict is detected in the MCC, it triggers prefrontal regulatory signals aimed at biasing competition to favor task-relevant cues over potential sources of distraction, such as the threat-distracters used in the present study (Miller and Cohen, 2001; Etkin et al., 2010). These biasing signals could be directed at the visual cortex (Miller and Cohen, 2001) or the amygdala (Etkin et al., 2011). At present, it remains unclear whether anxious individuals are less efficient at monitoring threat-related conflicts (Bishop et al., 2004; Etkin et al., 2010; Shackman et al., under review).

A third possibility is that anxious individuals' bias to allocate unnecessary storage to threat-distracters reflects a gating deficit. Consistent with recent computational models (Frank and O'Reilly, 2006; Moustafa et al., 2008; Wiecki and Frank, 2010), the basal ganglia and dorsolateral prefrontal cortex (dlPFC) exhibit gating-like signals that are associated with reduced distracter-evoked activity in visual cortex and reduced storage of distracters in the PPC (Postle, 2005; McNab and Klingberg, 2008; Suzuki and Gottlieb, 2013) during emotionally-neutral working memory tasks. Furthermore, patients with lesions involving the basal ganglia (i.e., left putamen) show selective deficits in gating distracters when performing emotionally-neutral working memory tasks (Baier et al., 2010). Whether similar mechanisms support the regulation of threat-related or other emotionally-salient distracters is unknown. Nevertheless, robust projections from the amygdala to the basal ganglia (Freese and Amaral, 2009) suggest one way in which high levels of dispositional anxiety could promote threat's access to working memory. Functional interactions between the amygdala and dlPFC could provide an alternate pathway (Lim et al., 2009).

From a translational perspective, our results provide a framework for conceptualizing the intrusive and distressing thoughts, worries, and memories that are a central feature of anxiety and mood disorders, including generalized anxiety, obsessive compulsive, posttraumatic stress, and major depressive disorders (Beck et al., 2005; Nolen-Hoeksema et al., 2008). High levels of dispositional anxiety are associated with a similar pattern of dysregulated cognition (e.g., Eysenck, 1984; Eysenck and van Berkum, 1992). Inefficient filtering of threat-related information from working memory potentially explains many of these features. That is, once it resides in working memory, threat-related information could continue to elicit distress and maladaptively bias attention and action after it is no longer present in the external environment.

Importantly, this framework also provides a potential mechanistic explanation for the intrusive, distressing memories that are a hallmark of both dispositional anxiety and many disorders on the internalizing spectrum (Krueger and Markon, 2006). In particular, it has become clear that items can enter working memory via either perceptual encoding, as with the threat-related distracters used in the present study, or retrieval from long-term memory (Jonides et al., 2008). From this perspective, working memory reflects the temporary activation of recently perceived items or the temporary re-activation of representations stored in long-term memory (Oberauer, 2002; Jonides et al., 2008; Lewis-Peacock et al., 2012). This suggests that intrusive memories, such as those prominent in post-traumatic stress disorder, could result from problems preventing distressing long-term memories from gaining access to working memory.

On the basis of the present results and other data, we have proposed that the maladaptive profile of thoughts and behaviors exhibited by anxious individuals in the absence of overt threat could reflect a more fundamental deficit in controlling threat's access to working memory. Although it is clear that much work remains, this hypothesis provides a clear roadmap to the most fruitful avenues for understanding the neurocognitive mechanisms underlying these symptoms. In particular, as with any preliminary study, it will be important to replicate our findings using a larger sample (Yarkoni, 2009). Given that our conclusions were based on a convenience sample, it will be essential to test our hypothesis in high-risk and patient populations and to directly assess the degree to which threat-related filtering efficiency predicts differences in the severity or frequency of distressing thoughts and maladaptive behaviors. It may be that the presentation of gating deficits differs across internalizing disorders (Owens et al., 2012). Methodologically, it will be important to develop improved procedures for minimizing ocular artifacts, which led to substantial attrition in the present study and in other studies using similar paradigms (Sessa et al., 2011). Extending our approach to incorporate simpler cues (e.g., color patches or oriented bars) that have been aversively-conditioned may prove helpful in this regard and would have the added benefit of increasing integration with the large body of cognitive neuroscience research and theory developed around such stimuli (see Owens et al., 2012 for a related application).

Dispositional anxiety is an important risk factor for the development of anxiety, depressive, and other psychiatric disorders. The present study provides novel evidence that dispositional anxiety reflects a failure to adequately regulate the access of threat to working memory, the capacity-limited workspace that underlies adaptive, goal-directed behavior. These results set the stage for a more detailed understanding of the distressing thoughts and memories that afflict anxious individuals when threat is absent—a defining, but poorly understood feature of the internalizing spectrum of disorders. Future research aimed at clarifying the neural underpinnings of this regulatory deficit promises to enhance our understanding of the mechanisms that confer risk for the development of psychopathology.

## Author contributions

Daniel M. Stout conceptualized the study. Daniel M. Stout, Christine L. Larson, and Alexander J. Shackman designed the study. Daniel M. Stout collected data and performed data processing. Daniel M. Stout analyzed data. Daniel M. Stout, Alexander J. Shackman, and Christine L. Larson contributed to data interpretation. Daniel M. Stout and Alexander J. Shackman wrote the paper. Alexander J. Shackman and Daniel M. Stout created the figures and table. Christine L. Larson supervised the study. All authors contributed to revising the paper.

### Conflict of interest statement

The authors declare that the research was conducted in the absence of any commercial or financial relationships that could be construed as a potential conflict of interest.
